# Syk Kinase Inhibitors Synergize with Artemisinins by Enhancing Oxidative Stress in *Plasmodium falciparum*-Parasitized Erythrocytes

**DOI:** 10.3390/antiox9080753

**Published:** 2020-08-14

**Authors:** Ioannis Tsamesidis, Karine Reybier, Giuseppe Marchetti, Maria Carmina Pau, Patrizia Virdis, Claudio Fozza, Francoise Nepveu, Philip S. Low, Francesco Michelangelo Turrini, Antonella Pantaleo

**Affiliations:** 1 Department of Biomedical Sciences, University of Sassari, 07100 Sassari, Italy; johntsame@gmail.com (I.T.); gmarchetti@uniss.it (G.M.); paumc81@tiscali.it (M.C.P.); 2 UMR 152 Pharma-Dev, Université de Toulouse, IRD, UPS, 31000 Toulouse, France; karine.reybier-vuattoux@univ-tlse3.fr (K.R.); francoise.nepveu@univ-tlse3.fr (F.N.); 3 Department of Clinical, Surgical and Experimental Sciences, University of Sassari, 07100 Sassari, Italy; patriziavirdis70@gmail.com (P.V.); cfozza@uniss.it (C.F.); 4 Purdue Institute for Drug Discovery and Department of Chemistry, Purdue University, West Lafayette, IN 47907, USA; plow@purdue.edu; 5 Department of Oncology, University of Turin, 10126 Turin, Italy; francesco.turrini@unito.it

**Keywords:** *Plasmodium falciparum*, syk kinase inhibitors, artemisinin derivatives, hemichromes, oxidative stress, reactive oxygen species

## Abstract

Although artemisinin-based combination therapies (ACTs) treat *Plasmodium falciparum* malaria effectively throughout most of the world, the recent expansion of ACT-resistant strains in some countries of the Greater Mekong Subregion (GMS) further increased the interest in improving the effectiveness of treatment and counteracting resistance. Recognizing that (1) partially denatured hemoglobin containing reactive iron (hemichromes) is generated in parasitized red blood cells (pRBC) by oxidative stress, (2) redox-active hemichromes have the potential to enhance oxidative stress triggered by the parasite and the activation of artemisinin to its pharmaceutically active form, and (3) Syk kinase inhibitors block the release of membrane microparticles containing hemichromes, we hypothesized that increasing hemichrome content in parasitized erythrocytes through the inhibition of Syk kinase might trigger a virtuous cycle involving the activation of artemisinin, the enhancement of oxidative stress elicited by activated artemisinin, and a further increase in hemichrome production. We demonstrate here that artemisinin indeed augments oxidative stress within parasitized RBCs and that Syk kinase inhibitors further increase iron-dependent oxidative stress, synergizing with artemisinin in killing the parasite. We then demonstrate that Syk kinase inhibitors achieve this oxidative enhancement by preventing parasite-induced release of erythrocyte-derived microparticles containing redox-active hemichromes. We also observe that Syk kinase inhibitors do not promote oxidative toxicity to healthy RBCs as they do not produce appreciable amounts of hemichromes. Since some Syk kinase inhibitors can be taken daily with minimal side effects, we propose that Syk kinase inhibitors could evidently contribute to the potentiation of ACTs.

## 1. Introduction

Following entry into the bloodstream of its human host, *Plasmodium* sporozoites injected by an infected mosquito migrate to the liver and initiate the hepatic stage of the parasite life cycle by invading hepatocytes, within which they multiply and differentiate into schizonts containing thousands of hepatic merozoites. These merozoites are subsequently released into the blood where they initiate the erythrocytic stage by invading and replicating within red blood cells (RBCs), and they multiply ~18-fold every 48 h. During the course of their intra-erythrocyte life, the parasite ingests and digests about 70% of the host cell hemoglobin (Hb) with the obligate release of heme that must be converted into an insoluble crystalline form called hemozoin to prevent oxidative damage to the maturing parasite [1,2] and to maintain the colloid osmotic balance in the parasitized erythrocyte resisting premature lysis [3,4]. Suppression of hemozoin formation is thought to constitute a major mechanism of action of several anti-malaria drugs, including quinine, chloroquine, and mefloquine [5,6]. Artemisinin or one of its synthetic derivatives constitutes the other major component of most anti-malaria therapies [7,8]. Although the mechanisms of action of ACTs (artemisinin combination therapies) are still debated [9,10], most hypotheses invoke involvement of redox reactions in their cytotoxicity, since (i) the essential moiety in all therapeutically active forms of artemisinin is its endoperoxide ring [11], (ii) other components of common ACTs promote the accumulation of oxidative heme [12], (iii) erythrocyte mutations that increase oxidative stress within the parasitized RBC confer partial protection against malaria [13,14,15,16,17,18,19] (e.g., sickle cell, β-thalassemia, G6PDH (Glucose-6-phosphate dehydrogenase) deficiency, etc., and (iv) many other oxidizing drugs were found to exhibit antimalarial activity [20,21]. Taken together, these observations suggest that oxidative stress can be detrimental to the parasite, probably by creating an environment that is harmful to parasite maturation, proliferation, or egress. In addition to hemoglobin digestion, from the early stages of their development, parasites also lead to hemoglobin oxidation and partial denaturation products in the host cell [13,22]. In particular, *P. falciparum* intra-erythrocyte growth causes hemoglobin oxidation (methemoglobin) and its partial denaturation (hemichromes), characterized by iron binding to distal histidine in the heme pocket [23,24,25,26,27]. Interestingly, hemichromes (HMCs) are classified as reversible and irreversible. While low-spin state HMCs are poorly reactive, reversible HMCs can still be reduced to their high-spin functional, redox-active form [28]. HMCs bind with high affinity to the N-terminal of the cytoplasmic domain of band 3 [29,30,31,32,33,34], the major transmembrane erythrocyte protein which forms junctional complexes with the cytoskeleton, assuring mechanical stability of the membrane [35]. Following their binding to band 3, HMCs trigger the oxidation of the two cysteine residues (C201 and C317) lying in the band 3 cytoplasmic domain with the formation of two disulfide intermolecular cross-links between two band 3 molecules [22,36]. Notably, disulfide cross-linked band 3 dimers acquire the capability to dock Syk protein kinase, leading to the phosphorylation of band 3 Tyr 8 and Tyr 21 [37]. This event leads to the uncoupling of the oxidized phosphorylated band 3 from ankyrin, the major link between the membrane and cytoskeleton, facilitating the clustering of the band 3–hemichrome complex to form large aggregates known as Heinz bodies [32,38]. As expected from the essential role of the band 3–ankyrin complex in assuring membrane stability, those aggregates tend to protrude from the erythrocyte surface and are progressively released through membrane vesiculation [39]. As a matter of fact, membrane-bound HMC aggregates and circulating microparticles (MPs) were described in hemolytic diseases such as sickle cell anemia, thalassemias, G6PD deficiency, and malaria [40,41,42]. Notably, in all cited situations, those changes were attributed to the oxidative stress generated by denatured hemoglobin. As expected, the release of MPs from the membrane of parasitized RBCs determines the depletion of band 3 molecules, leading to pronounced membrane destabilization at the final stages of parasite development. We also demonstrated that counteracting band 3 depletion with Syk inhibitors impedes the final egress of merozoites [43,44,45]. Due to the recent emergence of malaria strains in Southeast Asia that exhibit resistance to ACTs [46,47,48,49], the quest for new anti-malarials that might synergize with artemisinins in preventing the spread of drug resistance is intensifying [50,51]. Recognizing that artemisinin needs to be converted in highly reactive carbon-centered radicals via endoperoxide cleavage and that HMCs containing redox active iron are generated in parasitized erythrocytes, we hypothesized that inducing the retention of HMCs through the inhibition of Syk kinase might both increase oxidative stress and accelerate the activation of artemisinin. In this possible mechanism, we also hypothesized that accumulation of reactive HMCs should also aggravate the oxidative stress caused by artemisinin catalyzing additional peroxidation reactions and inducing further HMC generation with a possible synergistic effect between Syk inhibitors and artemisinin. In this paper, we test multiple aspects of this hypothesis. We firstly show that Syk inhibitors promote increased accumulation of hemichromes within the infected RBC by preventing the release of hemichrome-enriched microparticles. We also observe that this accumulation is strongly augmented by co-administration of artemisinin, enhancing the oxidative stress within the infected erythrocyte. Upon demonstrating this phenomenon, as expected from our hypothesis, the combined effect of a Syk kinase inhibitor plus artemisinin is highly synergistic, creating a combination therapy with superior antimalarial activity to the two antimalarials administered alone (see graphical abstract of the proposed mechanism of action).

## 2. Material and Methods

Unless otherwise stated, all materials were obtained from Sigma-Aldrich, St. Louis, MO, USA.

### 2.1. Cultivation of Plasmodium falciparum-Infected RBCs (pRBCs)

Freshly drawn blood (Rh+) from healthy adults or from G6PD-deficient patients of both sexes was used. RBCs were separated from plasma and leukocytes by washing three times with RPMI (Roswell Park Memorial Institute) 1640 medium. *Plasmodium falciparum* laboratory strains Palo Alto, FCB1, and It-G (all mycoplasma-free) were used. Parasites were maintained in continuous culture using the method of Träger and Jensen [52]. Specifically, the parasitemia was routinely maintained around 5%, and parasites were synchronized by 5% sorbitol treatment as described by Lambros and Vanderberg [53]. For all experiments, mature parasites (shizonts and segmenters) after percoll separation [54] were added to washed RBCs and, 12 h after the infection (occurring within 6 h), the cultures were ready for the experimental procedures.

### 2.2. Ethics Statement

Healthy blood donors, all adults, provided written, informed consent before entering the study. The study was conducted in accordance with Good Clinical Practice guidelines and the Declaration of Helsinki. No ethical approval was requested as human blood samples were used only to sustain the parasites in in vitro cultures.

### 2.3. Drug Susceptibility Assays of Cultured Parasites

All Syk inhibitors were solubilized initially at 10 mM concentration in anhydrous DMSO (Dimethyl sulfoxide) and then serially diluted into anhydrous DMSO prior to the addition to malaria cultures. Untreated cultures were run in parallel with the same final concentration 0.01% (*v*/*v*) of DMSO as the drug-treated cultures. Cultures at the ring stage of *P. falciparum* were treated at 12 h post infection, from 3 to 24 h, with the indicated concentrations of dihydroartemisinin (DHA), artesunate (AS), or artemether (ATH) combined with the desired concentration of one of the following Syk inhibitors: P505-15 (Selleckchem); R406 (Calbiochem, Darmstadt, Germany), entospletinib (Selleckchem), Syk inhibitor II (henceforth abbreviated as SYK II), piceatannol, or imatinib (Santa Cruz Biotechnology).

### 2.4. Preparation of Iron Chelator Deferasirox

Deferasirox (DFX) (Med Chemexpress) was dissolved in DMSO as a 1 mM stock solution and diluted into culture medium to achieve a final DMSO concentration of 0.001% (*v*/*v*). Stock solutions were then filtered through a Swinnex Millipore filter (pore size 0.2 µm) for sterilization prior to addition to malaria cultures.

### 2.5. Isobologram Preparation and Combination Index (CI) Measurement

Microsoft Excel was used to plot parasite counts following treatment with different combinations of the above drugs. To characterize the nature of the interaction (i.e., synergy, additivity, antagonism) between any two drugs, the experiments were performed at the IC_50_ (half maximal inhibitory concentration) concentrations obtained in in vitro experiments in pRBCs as previously described. The desired inhibitors were plotted on the *x*- and *y*-axes, and the line of additivity was constructed by connecting the two points defining the IC_50_ values of the two monotherapies. As defined in the quantitative combination index theorem of Chow–Talalay [55,56], experimental data points located below, on, or above the additivity line are interpreted to indicate synergy, additivity, or antagonism, respectively.

### 2.6. Assessment of Parasitemia by Light Microscopy

To evaluate the parasitemia and the parasite stage, thin smears of infected parasite cultures were prepared at the indicated times and stained with Diff-Quick stain (Medion Diagnostics, CH). A minimum of 5000 cells were examined microscopically by three observers. The experiments were done at least in triplicate.

### 2.7. IC_50_ Measurement

To calculate the half maximal inhibitory concentrations of the different Syk inhibitors, either used as single drugs or in combination with others antimalarial drugs, we used ICEstimator software version 1.2. The program estimates IC_50_ values using a nonlinear regression function of the R software.

### 2.8. Preparation of Cells for Confocal Microscopy

RBCs and parasitized RBCs (pRBCs) treated for 12 h with P505-15 (0.5 µM) at ring stage were pelleted and washed twice in PBS containing 5 mM glucose and then fixed for 5 min in 0.5% acrolein in PBS (Phosphate-Buffered Saline). Cells were rinsed three times, then permeabilized in PBS containing 0.1 M glycine (rinsing buffer) plus 0.1% Triton X-100 for 5 min, and rinsed again 3× in rinsing buffer. To ensure complete neutralization of unreacted aldehydes, the cells were then incubated in rinsing buffer at room temperature for 30 min. After incubation, all nonspecific binding was blocked by incubation for 60 min in blocking buffer (PBS containing 0.05 mM glycine, 0.2% fish skin gelatin, and 0.05% sodium azide). Resuspended RBCs and pRBCs were allowed to adhere to polylysine-coated cover slips, after which the cover slips were mounted with Aqua-Mount (Lerner Laboratories, New Haven, CT). The autofluorescence of hemichromes was visualized by exciting at 488 nm and observing the emission in the 630–750 nm range (pRBCs stained for nucleic acid by DAPI (4′,6-Diamidino-2-Phenylindole). Samples were imaged using a Bio-Rad MRC1024 (Bio-Rad) confocal microscope equipped with a 60 × 1.4 numerical aperture oil immersion lens.

### 2.9. Hemichrome Analysis

RBCs and pRBCs were incubated for 3, 6, 12, and 24 h with Syk inhibitors (Imatinib, R406, and P505-15) (0.5 µM) with/without DHA (0.5 nM). Cells were washed with cold PBS, and hypotonic membranes were prepared at 4 °C as previously described [57]. To solubilize the HMCs and to dissociate the cytoskeletal proteins, membranes were treated with 130 mM NaCl, 10 mM HEPES, 1mM EDTA (Ethylenediaminetetraacetic acid), and 1.5% C12E8 [57] and incubated under stirring (1400 rpm) at 37 °C for 15 min (Eppendorf ThermoMixer^®^ C.). To eliminate insoluble aggregates and debris originated from the parasite, detergent-treated membranes were centrifuged for 5 min at 20 °C, 15,000× *g*. To isolate the high-molecular-weight protein aggregate containing HMCs, the supernatant was loaded on a Sepharose CL6B column [57], chromatographic fractions were screened by visible spectroscopy, the fractions were characterized by the absorption spectrum of HMCs, and lack of the absorption peaks of Hb at 520 and 280 nm was collected for the quantitative measurement of HMCs and characterization of its components.

HMCs were quantified in the high-molecular-weight fraction by visible spectrometry using the following equation:

−133 × Abs577 − 144 × Abs630 + 233 × Abs560(1)

expressed as nmol/mL of solubilized membranes [58,59].

### 2.10. Hemoglobin Release Quantification

Following centrifugation at 1000× *g*, hemoglobin concentration was measured in the culture supernatant as previously described [60].

### 2.11. Electron Paramagnetic Resonance (EPR) Measurements

The detection of free radicals was carried out using *N*-*tert*-butyl-α-phenylnitrone (PBN) as a spin trap. PBN (1 M stock solution in DMSO) was added to normal RBCs, RBCs treated with 400 µΜ phenylhydrazine (PHZ), or parasitized (parasitemia 5%) pelleted red blood cells (from 2% hematocrit culture), and the volume was adjusted with PBS after addition of dihydroartemisinin (200 µM in DMSO) or Syk inhibitor (P505-15) (0.5 µΜ) and different concentrations (100–400 µΜ) of Deferasirox when needed. The solution was then transferred into a flat quartz cell (FZKI160-5 × 0.3 mm, Magnettech, Berlin, Germany) for EPR analysis. EPR spectra were obtained at room temperature (RT) using the X-band on a Bruker EMX-8/2.7 (9.86 GHz) equipped with a gaussmeter (Bruker, Wissembourg, France) and a high-sensitivity cavity (4119/HS 0205). WINEPR and SIMFONIA software (Bruker, Wissembourg, France) were used for EPR data processing and spectrum simulation. Typical scanning parameters were as follows: scan number, 5; scan rate, 1.2 G/s; modulation frequency, 100 kHz; modulation amplitude, 1 G; microwave power, 20 mW; sweep width, 100 G; sweep time, 83.88 s; time constant, 40.96 ms; and magnetic field, 3460–3560 G. The intensity of the EPR signal was calculated by double integration of the EPR Signal.

### 2.12. ROS Analysis

Glutathione (GSH) and *N*-acetyl-l-cysteine (NAC) were purchased from Sigma (France).

For the detection of intracellular reactive oxygen species (ROS) levels, we employed the cell-permeable ROS-sensitive probe 2′,7′-dichlorodihydrofluorescein diacetate (CM-H_2_DCFDA) which fluorescens at 520 nm (λex = 480 nm) upon oxidation. Oxidation of CM-H_2_DCFDA (prepared as a 0.5 mM stock solution in DMSO) in RBCs was monitored by measurement of the fluorescence of the desired RBC suspensions (0.2% Hematocrit) in 96-well black-walled microplates (Corning^®^, Sigma Aldrich) using an SAFAS Xenius (Monaco). The relative fluorescence is expressed as “% maximal emission” as determined with the software “Xenius”, where maximal emission was defined as the fluorescence emission obtained following addition of 3 mM H_2_O_2_.

### 2.13. Isolation and Characterization of Microparticles (MPs)

RBCs and pRBCs at ring stage were treated with P505-15 for 12 h. Supernatants containing the RBC-derived MPs were then collected and centrifuged at 25,000× *g* for 10 min at 4 °C to eliminate spontaneously formed red cell ghosts and cell debris. To isolate MPs, the supernatant was then centrifuged at 100,000× *g* for 3 h at 4 °C; the morphology and the size of isolated MPs were documented by confocal microscopy through the characteristic autofluorescence of HMCs (excitation: 488 nm; emission: 630–700 nm) as previously described [39,61].

The presence of HMCs in MPs was further confirmed by visible spectrometry analysis (characteristic absorption spectrum of HMCs [57] and total absence of the absorption peaks of Hb at 520 and 580 nm). Proteins composition of MPs was documented by mass spectrometry analysis as previously described; briefly, MPs obtained from 2 mL of pRBCs were pelleted and solubilized with 2% SDS, solubilized proteins were separated by SDS-PAGE, and, following staining with colloidal Coomasie blue, electrophoretic bands were excised and identified by high-resolution MALDI-TOF peptide mass fingerprinting.

MPs were characterized and quantified by FACS (flow cytometry) analysis as described [44] utilizing a BD FACSCanto^™^ A flow cytometer (Becton Dickinson Biosciences, San Jose, CA, USA). The RBCs and pRBCs were excited with blue (488 nm, air-cooled, 20 mW solid state) and red (633 nm, 17 mW HeNe) laser light. Data from at least 50,000 events were acquired and analyzed with FACSDiva TM software (Becton Dickinson Biosciences, San Jose, CA, USA). MPs from RBCs and pRBCs were identified by their fluorescence (Glycophorin-A positive cells) and quantified with BD Trucount^™^ beads (Becton Dickinson Biosciences, San Jose, CA, USA). The MPs localized within the platelet region (R1) were distinguished from platelets (CD61-FITC positive) by their glycophorin-A-positive/CD61-negative response. The number of BD Trucount^™^ beads was counted, and the absolute numbers of MPs were then calculated with the following formula: (No. of glycophorin A positive events/No. of beads collected) × BD Trucount^™^ concentration × dilution factor. MPs and platelets were incubated with 20 µL of PBS-G (PBS containing 2 mM glucose) containing 3 µL of PE-conjugated PE Mouse Anti-Human glycophorin A Clone GA-R2 (HIR2) (Becton Dickinson Biosciences, San Jose, CA, USA) monoclonal antibody, alone or with 5 µL of FITC-conjugated Mouse Anti-Human CD61 Clone VI-PL2 (Becton Dickinson Biosciences, San Jose, CA, USA) monoclonal antibody for 30 min at RT (room temperature) in the dark. The MPs were diluted in 400 µL of PBS-G–paraformaldehyde 1% and transferred to flow cytometer tubes preloaded with a known density of fluorescent BD Trucount^™^ beads (Becton Dickinson Biosciences, San Jose, CA, USA, Catalog No 340334) and quantified.

MPs were also quantified measuring their band 3 content by Western blot analysis as previously described [61].

### 2.14. Statistical Analysis

We performed comparisons with the use of Student’s *t*-test (two-tailed) using the SPSS version 22.0 Statistical package. Descriptive statistics are presented as mean ± standard deviation. Additionally, an independent sample *t*-test was used to compare between means. In all statistical analyses, the level of significance (*p*-value) was set as * *p* < 0.05, ** *p* < 0.001.

## 3. Results

### 3.1. Effect of Syk Inhibitors on Accumulation of Hemichromes in Parasitized RBCs

**Figure 1:**
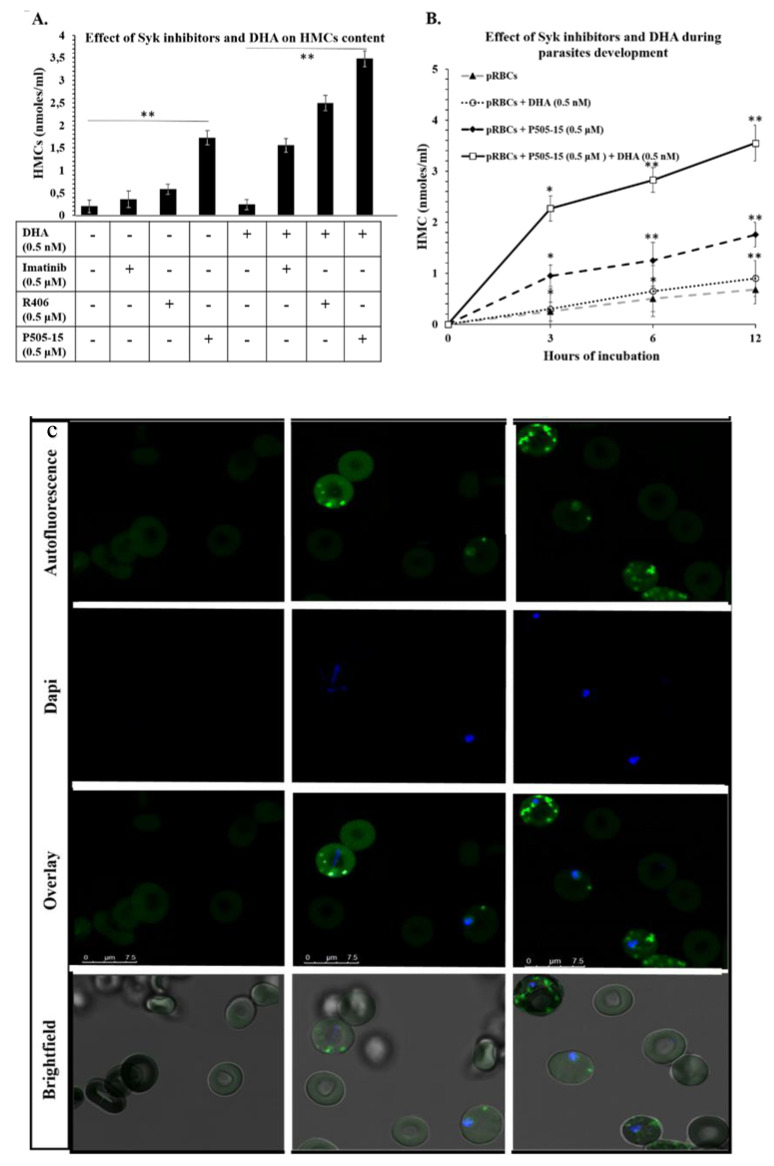
(**A**) Effect of parasitized red blood cell (pRBC) treatments with Syk inhibitors (imatinib, R406, and P505-15) (0.5 µM) and dihydroartemisinin (DHA) (0.5 nM) on the accumulation of HMC after 12 h of incubation. (**B**) Time course of hemichrome (HMC) accumulation contained in pRBCs treated with the representative Syk inhibitor, P505-15 (0.5 µM) and/or DHA (0.5 nM) for 3, 6, and 12 h. Data are the average of five independent experiments ± SD. Significant differences to untreated pRBCs at * *p* < 0.05; ** *p* < 0.001. (**C**) Confocal Images of HMCs contained in pRBCs untreated and treated for 12 h with P505-15 (0.5 µM). HMCs were visualized by their autofluorescence at 488 nm (excitation)/630–750 nm (emission). Images were acquired using the same magnification with a Leica TCS SP5 X (Leica Microsystems, Germany) confocal microscope equipped with a 60 × 1.4 numerical aperture oil immersion lens. The scale bar in the figure is 7.5 µm.

To test the hypothesis that Syk tyrosine kinase inhibitors might increase the hemichrome (HMC) content of malaria-infected erythrocytes (pRBCs) by blocking release of hemichrome-enriched microparticles, we firstly measured the HMC concentrations in pRBCs in the presence and absence of a variety of Syk inhibitors. As shown in Figure 1A, although untreated pRBCs accumulated only low levels of HMCs, incubation with different Syk inhibitors characterized by different IC_50s_ on isolated Syk (P505-15: 1 nM, R406: 41 nM, imatinib: 5 µM) increased accumulation of HMCs, and this accumulation correlated roughly with the potency of the specific Syk inhibitor. Because these inhibitors have no detectable effect on uninfected RBCs, we conclude that Syk inhibitors can enhance accumulation of hemichromes in infected RBCs without promoting hemichrome formation or accumulation in healthy erythrocytes. Next, to determine whether addition of dihydroartemisinin (DHA) might further enhance Syk inhibitor-promoted accumulation of HMCs, the same experiment was repeated using a very low concentration of DHA (0.5 nM) to minimize the cytotoxic effects on *P. falciparum*. This low concentration of DHA did not cause an increase of HMCs in RBCs or in pRBCs. On the contrary, DHA in combination with Syk inhibitors caused a consistent increase of HMC content in pRBCs. No effect on HMC accumulation was measurable in control RBCs (Figure S1, Supplementary Materials). Our motivation for employing such a low concentration of DHA was to obtain an initial indication of possible synergy between DHA and a Syk inhibitor, since the concentration of DHA employed in this study was found to promote no hemichrome formation by itself (Figure 1) and no measurable anti-plasmodial effect. In this regard, it should be noted that, within 12 h of treatment, no morphological changes of parasites were observed with any treatment. On the contrary, the combination of DHA and Syk inhibitor caused a moderate delay of maturation after 24 h. To minimize the bias that may derive from the loss of viability of parasites, data presented in Figure 1 were obtained within 12 h of treatment.

As predicted from our hypothesis, co-administration of the low dose of DHA (0.5 nM) further augmented the hemichrome content of Syk inhibitor-treated RBCs (Figure 1A). Moreover, as shown in Figure 1B, this aggravated accumulation of hemichromes likely begins almost immediately following drug addition, since the pRBC content of hemichromes is prominently increased by 3 h post administration. As Syk inhibitors were added to cultures 12 h post infection, we could notice that hemichrome accumulation was already enhanced at the ring stage. This observation is in accordance with previous reports showing that pro-oxidant hemoglobinopathies such as G6PD deficiency, hemoglobins C and S, and thalassemias can promote hemichrome production in the first half of parasite development [13,23,24,26,34,55]. Taken together, these data suggest that Syk inhibitors and DHA may synergize in enhancing accumulation of reactive hemichromes in pRBCs. We then studied the effect of Syk inhibitors in the presence and absence of DHA during the earlier stages of parasite development. Figure 1B shows the time-dependent accumulation of HMCs in untreated pRBCs and in pRBCs treated with Syk inhibitor (P505-15) in the presence and absence of DHA. Although treatment with DHA had little effect on HMC concentrations at any maturation stage, P505-15 exerted considerable impact on HMC accumulation during all early stages of parasite maturation (from early ring stage cultures to trophozoites), and this effect was further enhanced by co-administration of a low dose (0.5 nM) of DHA. Treatment with the other Syk inhibitors (R406 and imatinib) caused similar degrees of HMC accumulation, both in the presence and in the absence of DHA. Indeed, by imaging the autofluorescence of HMCs upon excitation at 488 nm (λ_em_ = 630–700 nm), the absence of HMCs in healthy RBCs, their low abundance in pRBCs, and their strong accumulation in P505-15-treated pRBCs could be visually confirmed (Figure 1C). HMCs were also measured following their semi-selective extraction from erythrocyte membranes by mild detergent treatment followed by size-exclusion purification to isolate the high-molecular-weight aggregate (>2 × 10^6^ kDa) containing HMCs. HMCs were quantified according to their spectral characteristics [28,55]; notably, in the high-molecular-weight fraction, no free oxyHb was detectable (lack of absorption peaks at 488 and 520 nm). The purity of HMCs contained in the high-molecular-weight fraction, measured as a fraction of total protein concentration, was higher than 80%.

### 3.2. Effect of Syk Inhibitors on MP Production

**Figure 2:**
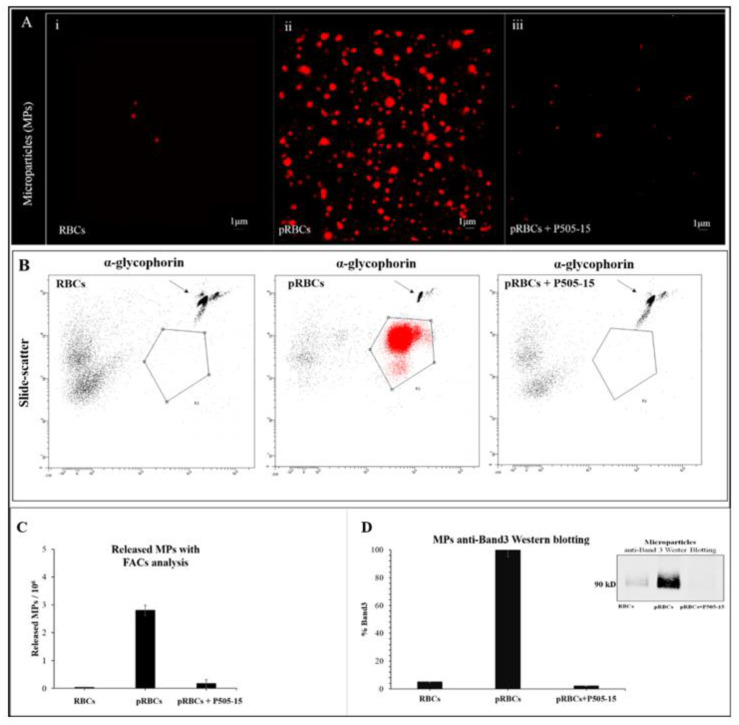
(**A**) Confocal Images of isolated microparticles (MPs) contained in pRBCs untreated and treated for 12 h with P505-15 (0.5 µM). MPs were visualized by their autofluorescence at 488 nm (excitation)/630–750 nm (emission). Images were acquired using the same magnification with a Leica TCS SP5 X (Leica Microsystems, Wetzlar, Germany) confocal microscope equipped with a 60 × 1.4 numerical aperture oil immersion lens. The scale bar in the figure is 1 µm. (**B**) Representative flow cytometric density plot of MPs in supernatant collected from RBCs, pRBCs, and P505-15-treated pRBCs. MPs were identified as glycophorin A-positive events (R1). The number of MPs was quantified using the known density of fluorescent trucount^TM^ beads (arrows). (**C**) Graphical representation of flow cytometric density plot of MPs. (**D**) MP anti-band 3 Western blotting.

Next, to test the hypothesis that hemichromes are discharged from pRBCs by blebbing off hemichrome-enriched microparticles (MPs), following a first centrifugation step to eliminate erythrocyte ghosts and debris, we pelleted the MPs at 100,000× *g* from the supernatants of both healthy and parasitized RBC suspensions. Isolated MPs were then resuspended and examined by confocal microscopy exploiting the fluorescence of HMCs upon excitation at 488 nm (λ_em_ = 630–700 nm). As shown in Figure 2A, the supernatant collected from healthy RBCs was essentially void of MPs. In contrast, the supernatant from untreated pRBCs was found to contain considerable hemichrome-loaded MPs, i.e., as evidenced by the autofluorescence of the MPs. More importantly, the supernatant from Syk inhibitor-treated (P505-15) pRBCs displayed essentially the same level of autofluorescent MPs as seen in suspensions of healthy RBCs. The presence of high-molecular-weight protein aggregates in MPs was confirmed utilizing the same method used for pRBC membranes (see previous paragraph). Spectral analysis also revealed the absence of oxyHb, confirming a strong analogy between the high-molecular-weight HMC aggregates isolated from pRBC membranes and MPs. As previously observed [20], MPs isolated from pRBCs supernatants were relatively homogeneous with a diameter lower than 1 µm; moreover, the morphology and the dimensions of the fluorescent aggregates observed in the MPs are compatible with HMC aggregates observed in the pRBC membranes. MPs were characterized and quantified by FACS utilizing anti-glycophorin antibody to label RBC membranes. Western blot analysis using anti-band 3 antibody further confirmed the variations of RBC membrane-derived MPs (Figure 2B–D).

To substantiate the role of oxidative stress in MP generation, we treated pRBCs with ROS scavengers reduced glutathione and *N*-acetyl-l-cysteine. As shown in the fifth figure (C,D), those compounds inhibited ROS production and the release of MPs. The relatively high concentrations needed to exert the observed inhibition are plausibly due to the limited membrane permeability of those molecules.

### 3.3. Hemichromes Accumulation and ROS Production

**Figure 3:**
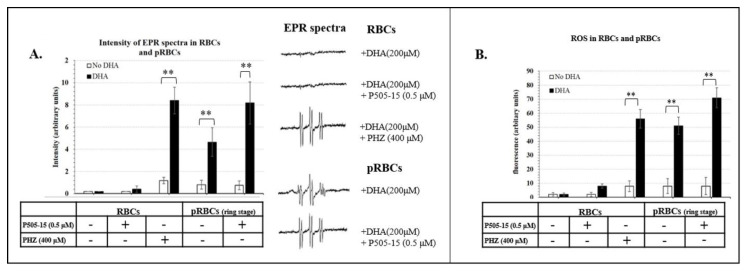
(**A**) Intensity of electron paramagnetic resonance (EPR) spectra (arbitrary units) in the presence of *N*-*tert*-butyl-α-phenylnitrone (PBN) spin trapping agent in RBCs treated with phenylhydrazine (PHZ) (400 µΜ) with/without DHA (200 µΜ). RBCs and pRBCs treated with/out P505-15 (0.5 μΜ) for 24 h with/without 10-min incubation of DHA (200 µΜ). (**B**) The same experiments mentioned above (**A**) were performed using the fluorescent probe 2′,7′-dichlorodihydrofluorescein diacetate (CM-H_2_DCFDA), a cell-permeable indicator of reactive oxygen species (ROS). Data are the average ± SD of five independent experiments. Significant differences between no-DHA RBCs and pRBCs at ** *p* < 0.001.

The next element of our hypothesis was that enhanced accumulation of hemichromes in Syk inhibitor- and DHA-treated pRBCs should increase the oxidative stress within the pRBCs. To test this step in the hypothesis, we quantitated the level of free radicals generated within the RBCs by EPR using a spin trap, α-phenyl-*N*-*tert*-butylnitrone (PBN), to stabilize any generated free radicals. The six-lined spectra, characteristic of the PBN adduct formed after radical trapping in different samples, are presented in Figure 3A. Simulation of the spectra yielded the following parameters: *g* = 2.0059, a_N_ = 14.9 G, and a_H_ = 3.3 G, indicating that a hydroxyl radical was generated and transformed into a CH_3_ radical after reaction with DMSO. Importantly, little or no signal was recordable in either control RBCs or healthy RBCs treated with DHA, P505-15, or both together, i.e., indicating that the combination of DHA + Syk inhibitor does not induce significant oxidative stress in healthy RBCs. In contrast, stimulation of HMC formation in healthy cells by treatment with phenylhydrazine (PHZ) was found to enable the DHA + Syk inhibitor combination to potently induce free radical formation, i.e., suggesting that HMCs must be present to catalyze the antimalarial-induced formation of free radicals. Consistent with these results, although only a low-intensity EPR signal was observed in untreated pRBCs (ring stage), this signal was significantly enhanced upon brief incubation (10 min) with 200 µM DHA (this high concentration was chosen due to the short time of incubation) and then further strengthened by addition of P505-15 (*p*-value = 0.028) (Figure 3A). Since very similar results were obtained when the ROS-sensitive fluorescent probe, CM-H_2_DCFDA (*p*-value = 0.022), was employed to quantitate oxidative stress (Figure 3B), we conclude that, in the presence of HMCs, induced by phenylhydrazine or parasite growth, intra-erythrocyte oxidative stress can be greatly increased by the addition of Syk inhibitors plus DHA.

**Figure 4:**
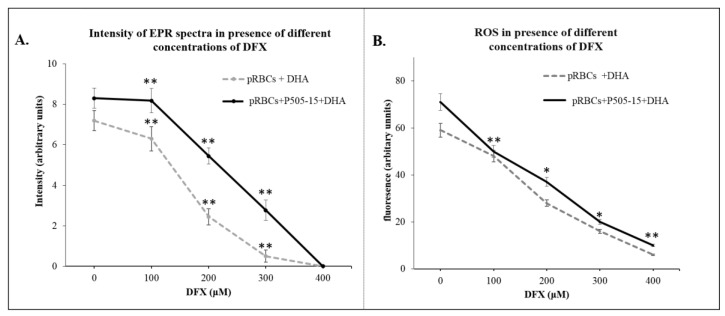
(**A**) Intensity of EPR spectra (arbitrary units) in the presence of PBN spin trapping agent in pRBCs treated with/without P505-15 (0.5 μΜ) for 24 h with/without 10-min incubation with DHA (200 µΜ) and with different concentrations (100–400 µM) of Deferasirox (DFX). (**B**) The same experiments mentioned above (**A**) were performed using the probe CM-H_2_DCFDA. Data are the average ± SD of five independent experiments. Significant differences between untreated DHA-RBCs and pRBCs at * *p* < 0.05, ** *p* < 0.001.

Figure 4A, chelation of the heme iron significantly decreased the intensity of the free radical signal (*p*-value = 0.019), indicating the requirement of accessible iron in the production of activated DHA and oxidative free radicals in the parasitized RBC.

**Figure 5:**
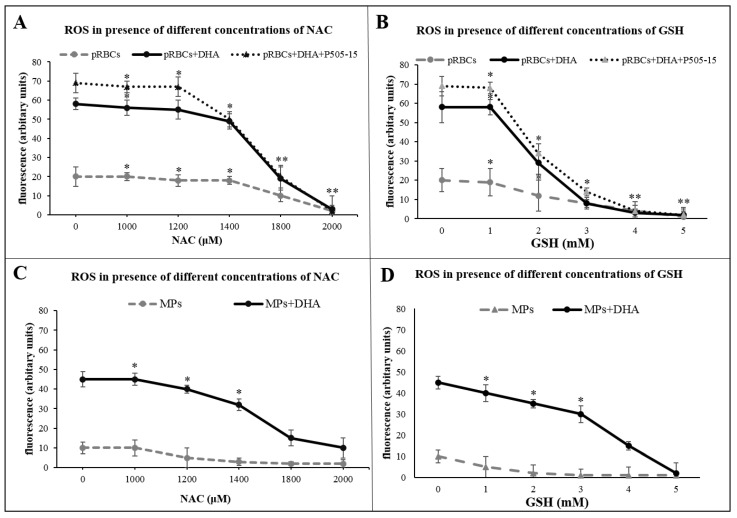
ROS production was performed using the probe CM-H2DCFDA in pRBCs pretreated with/without P505-15 (0.5 μΜ) and DHA (200 μΜ) and their MPs for 24 h at different concentrations of *N*-acetyl-l-cysteine (NAC) (**A**,**C**) and GSH (Glutathione) (**B**,**D**). Significant differences between untreated DHA-RBCs/MPs and pRBCs/MPs at * *p* < 0.05, ** *p* < 0.001.

These data suggest that the availability of reactive iron in hemichrome-rich pRBCs but essentially absent from healthy RBCs is essential for the catalysis of activated DHA and ROS production. As evidenced in the previous paragraph, ROS scavengers decreased the production of ROS in pRBCs (Figure 5A,B) and MPs released from pRBCs (Figure 5C,D).

### 3.4. Evaluation of Synergy between Syk Inhibitors and Artemisinins in Suppression of Parasitemia

**Figure 6:**
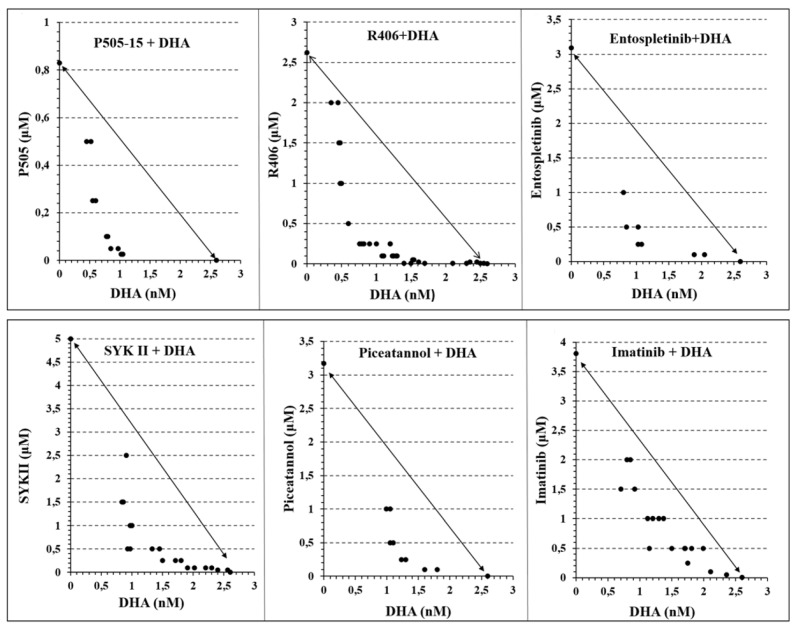
Isobolograms showing the interactions between Syk Inhibitors (P505-15, R406, entospletinib, SYK II, piceatannol, and imatinib) and dihydroartemisinin (DHA), after 24 h of incubation in the *P*. *falciparum* Palo Alto strain. Synchronized *P. falciparum* cultures were treated for 24 h with different concentrations (from 0.05 to 2.5 µM) of different Syk inhibitors (P505-15, R406, entospletinib, SYK II, piceatannol, and imatinib) in combination with different concentrations of DHA (from 0.6 to 10 nM) at the ring stage. IC_50_ concentrations of all the drug combinations were plotted in isobolograms to determine synergy, additivity, or antagonism.

The previous paragraphs suggest that DHA and Syk inhibitors act synergistically to induce the formation of ROS in pRBCs. To determine whether this synergistic production of ROS might translate into synergistic suppression of parasitemia, we employed the combination index theorem of Chow–Talalay [55,56] to quantitate the synergy/antagonism between DHA and Syk inhibitors in eliminating *P. falciparum* from cultures of fresh human blood. As noted in Section 3, experimental data points in the required isobolograms that lie below the diagonal line indicate synergy, while those that lie on the diagonal demonstrate additivity, and those that reside above the line are interpreted to indicate antagonism. To construct these isobolograms, ring-stage pRBCs (12 h post infection) were treated with increasing concentrations of the desired drugs for 24 h, and the residual parasitemia was quantitated to determine the IC_50_ value of each Syk inhibitor at the indicated concentration of artemisinins. As seen in Figure 6, regardless of the Syk inhibitor employed, all inhibitors synergized with DHA in suppressing parasitemia.

Moreover, as noted from their quantitative combination index values (Table 1), the strength of the observed synergies corresponded approximately with the potencies of the Syk inhibitors (i.e., combination index (CI) values were 0.42 for P505-15 and R406, followed by entospletinib and SYK II at 0.51, and then piceatannol and imatinib at 0.56 and 0.73, respectively), and similar strong synergy was also observed when other forms of artemisinins were evaluated (Table S1, Supplementary Materials).

To add evidence to the role of oxidative stress mediated by HMCs in the mechanism leading to the synergistic interaction between Syk inhibitors and artemisinin, we conducted experiments growing *P. falciparum* in G6PD-deficient RBCs that, due to their reduced capability to produce NADPH (Nicotinamide adenine dinucleotide phosphate), are known to maximize the effects of oxidative stress, promoting the formation of HMCs both in vitro and in G6PD-deficient patients following ingestion of redox-active drugs or fava beans [30,62,63]. As previously described [34], parasites display the same growth rate in control and G6PD-deficient RBCs. In G6PD-deficient RBCs, we observed an increment in HMC content following DHA and Syk inhibitor treatment (Figure S2 Supplementary Materials). In addition, in G6PD-deficient RBCs, we measured a significant (*p* < 0.01) decrease in IC_50_ for DHA (from 2.60 nM to 1.75 nM) and a variable increase in synergistic interaction between DHA and Syk inhibitors. The CI shifted from 0.42 to 0.36 for P505-15 and from 0.73 to 0.33 for imatinib. The change in CI was significant (*p* < 0.01) only for the less specific Syk inhibitor imatinib, possibly indicating that potent Syk inhibitors are capable of determining the maximal synergistic effect on DHA (Figure S3, Supplementary Materials).

It was also important to notice that treatments with DHA + Syk inhibitors did not cause any increase in HMCs in non-parasitized G6PD-deficient RBCs, confirming that this combination does not cause oxidative injury to RBCs. To further investigate this relevant issue, we measured hemolysis as the percentage of total Hb contained in RBCs released in the supernatant following 24 h of treatment with DHA (10 nM) + P505-15 (250 nM). In untreated control and G6PD-deficient RBCs, we could not observe a significant difference in hemolysis (1.21% ± 0.76% and 1.66% ± 0.53%, respectively); following 24 h of treatment, we observed a non-significant increase in hemolysis both in control and in G6PD-deficient RBCs (2.43% ± 1.76% and 3.06% + 1.53%, respectively).

**Figure 7:**
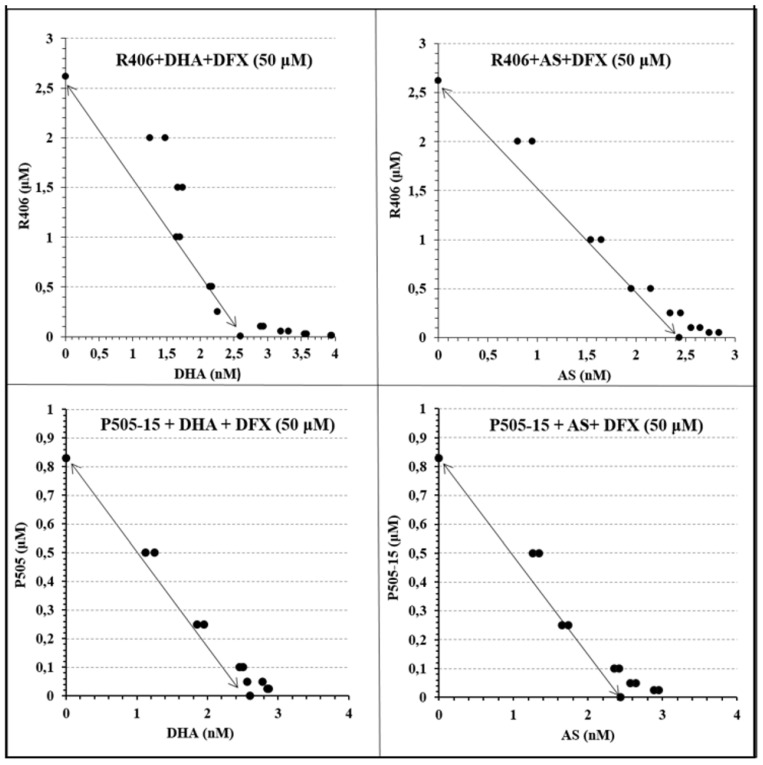
Isobolograms showing the interactions between Syk Inhibitors (P505-15 and R406) and dihydroartemisinin (DHA) and artesunate (AS) in combination with the iron chelator Deferasirox (DFX), after 24 h of incubation in the *P*. *falciparum* Palo Alto strain. Synchronized *P. falciparum* cultures were treated for 24 h with different concentrations (from 0.05 to 2.5 µM) of representative Syk inhibitors (P505-15 and R406) in combination with different concentrations of DHA and AS (from 0.6 to 10 nM) using a fixed concentration (50 µM) of the iron chelator Deferasirox (DFX) at the ring stage.

Because chelation of iron by DFX was found to block the production of ROS by DHA + Syk inhibitor (Figure 6), we then predicted that DFX might similarly prevent the synergistic elimination of parasitemia by DHA + Syk inhibitor. As shown in Figure 7 and Table 2, this prediction was indeed realized.

Thus, regardless of whether dihydroartemisinin or artesunate was combined with P505-15 or R406 (Syk inhibitors), addition of DFX moved all data points back to the diagonal line, indicating the elimination of synergy between artemisinin and Syk inhibitor. These data argue that reactive iron and ROS production are critical to creation of the potent synergy between these two classes of antimalarial drugs.

**Figure 8:**
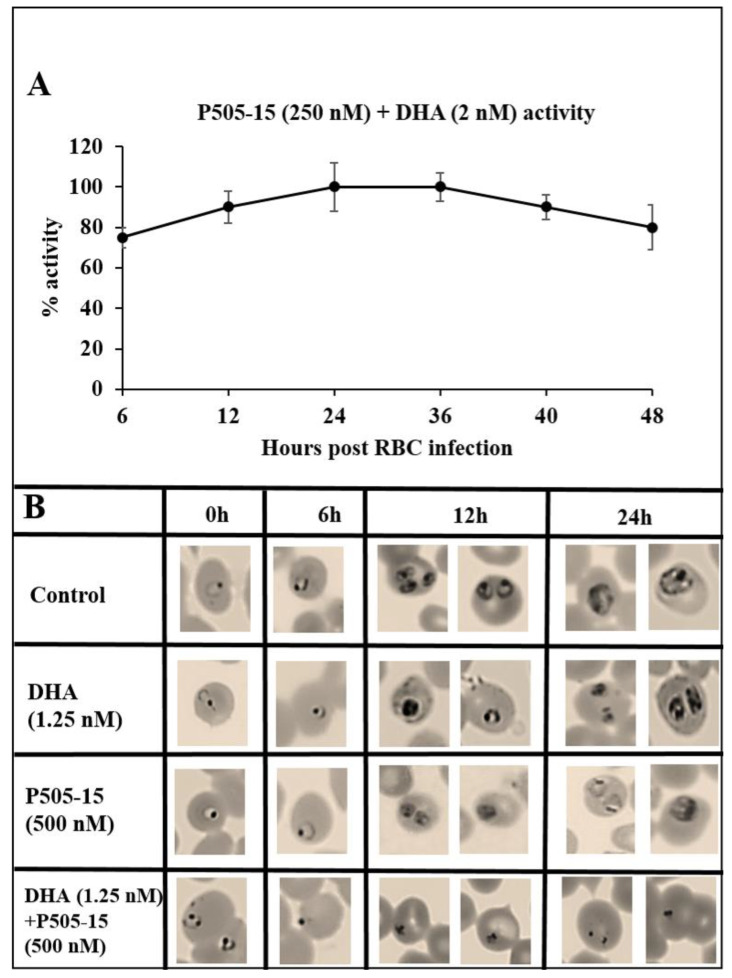
(**A**) Stage dependency of the efficacy (6, 12, 24, 36, 40, and 48 h) expressed as relative activity at one fixed dosage of DHA + P505-15 (2 nM + 250 nM). Relative activity of DHA + P505-15 (2 nM + 250 nM) added at 6, 12, 24, 36, 40, and 48 h of incubation. Values are expressed as percentage of DHA + P505-15 activity (treatment time at 24 h post infection) measured as % activity. Data are the average of five experiments ± SD. (**B**) Morphological changes in *P. falciparum* induced by Syk inhibitor (P505-15) in combination with a fixed concentration (1.25 nM) of dihydroartemisinin after 6, 12, and 24 h of treatment. Representative images of selected damaged parasites at 6, 12, and 24 h of treatment in control and drug-treated cultures selected from Diff-Quik^®^ fix-stained thin blood films. The micrographs were obtained using a Leica DM IRB microscope equipped with a 100× oil planar apochromatic objective with 1.32 numeric aperture and a DFC420C camera and DFC software version 3.3.1 (Leica Microsystems, Wetzlar, Germany). The scale bar in the figure is 7.5 µm.

Finally, to determine whether the combined action of Syk inhibitor plus artemisinin on *P. falciparum* survival might depend on the stage of parasite maturation, we compared the effects of the combination therapy with each monotherapy at different stages of parasite development. Figure 7 shows that, upon adding DHA + P505-15 (2 nM + 250 nM) at different stages of parasite development, the maximal activity was observed between 12 and 36 h post infection corresponding to ring and trophozoite stages. The data in Figure 8B reveal that none of the drugs have a measurable impact on pRBC morphology at 6 h post infection, after which the monotherapies (i.e., DHA or Syk inhibitor alone) display detectable but comparatively mild effects (few pycnotic cells (less than 2%)) on pRBC morphology.

In contrast, by 12 h post infection, the combination therapy is seen to exert a major influence on growth progression and pRBC morphology, essentially blocking the normal pathway of parasite maturation. While other explanations of the synergistic potency of the combination therapy can be envisioned, the data are very consistent with the ability of DHA to enhance the oxidative stress arising from hemichromes accumulated in pRBCs and with the capacity of Syk inhibitors to greatly augment this oxidative stress by preventing the discharge of these oxidative hemichromes in HMC-enriched microparticles.

## 4. Discussion

Based on all the above data, we wish to propose a mechanism for the effects of artemisinins and Syk inhibitors on *P. falciparum* parasitemia. Following invasion of an erythrocyte, the parasite must digest hemoglobin in order to multiply ~18-fold during its 48-h intra-erythrocyte life cycle [19]. Parasite growth is accompanied by the production of ROS leading to hemoglobin oxidation and the formation of the hemichromes [13,64,65,66], which are known to be redox-active compounds [28,67,68]. In order to reduce this oxidative toxicity, the parasite promotes erythrocyte membrane weakening via activation of the phosphorylation of band 3 by Syk tyrosine kinase, which in turn enables the release of hemichrome-enriched MPs from the RBC membrane. In the absence of added drugs to enhance this oxidative stress, the parasite apparently copes with these oxidants from the pRBC. However, when a drug is added, it increases the pRBC’s oxidative stress, and the parasite’s ability to detoxify the added stress is exceeded as shown in Figure 3 and Figure 4, leading eventually to parasite death. Syk kinase inhibitors prevent the discharge of HMCs in membrane-encapsulated microparticles [44] and force the accumulation/retention of redox-active HMC forms, enhancing the oxidative stress within the pRBC and, therefore, activating artemisinins. On the other hand, we observed that artemisinin compounds strongly enhance the generation of HMCs in pRBCs. This apparently cooperative behavior between Syk kinase inhibitors and artemisinins may be at the basis of their synergistic anti-plasmodial interaction.

A mechanism to explain the observed synergy between artemisinins and Syk inhibitors is also implied in the data of Figure 3A,B, where artemisinins are not observed to induce oxidant production in healthy RBCs. We explain this difference between healthy RBCs and pRBCs by noting that redox-active hemichromes and/or traces of free heme released from hemichromes or from the parasite food vacuole are required to catalyze the production of pharmaceutically active artemisinin [69]. In addition, this mechanism could be relevant to determine the high selectivity of artemisinin for parasitized RBCs and, consequently, their effectiveness. The lower effectiveness of artemisinins observed in patients with hemoglobinopathies could, therefore, determine the loss of selectivity due to artemisinin activation in non-parasitized RBCs containing traces of hemichromes and/or free heme.

Based on this pathway, the activation and/or the potency of artemisinins must depend on the abundance of HMCs in parasitized RBCs, which in turn will depend on the ability of the parasite to eliminate these HMCs in discharged microparticles. The release of MPs containing hemichromes was already described in oxidized erythrocytes [70,71], in parasitized RBCs [20,44], and in different hematological diseases characterized by hemichrome formation such as thalassemias and G6PD-deficient RBCs following oxidant treatment [39,66]. Supporting the proposed mechanism of action, iron chelators and ROS scavengers suppressed the anti-plasmodial activity of the Syk inhibitor + artemisinin treatment. On the contrary, G6PD-deficient RBCs enhanced the synergistic combination between Syk inhibitor + artemisinin combination and HMC accumulation. Importantly, no hemichrome formation or hemolysis was observed in non-parasitized G6PD-deficient parasites treated by Syk inhibitors and artemisinins, evidencing the need of the additional ROS generated by malaria parasites to trigger a virtuous cycle between the accumulation of redox-active HMCs and the activation of artemisinin. Those data are also suggestive of a lack of hemolytic effect exerted by Syk inhibitor + artemisinin combination in normal and G6PD-deficient RBCs, although more specific tests will be conducted to exclude the pro-hemolytic activity of this combination.

## 5. Conclusions

In conclusion, the presented data highlight the complex interactions occurring among ROS production due to parasite metabolism, redox-active HMCs, and artemisinin activation, documenting the role of Syk inhibition as a key element to synergistically improve the activity of artemisinins. Since some Syk kinase inhibitors such as R406 can be administered for long periods with minimal side effects [72,73], we propose that Syk kinase inhibitors could contribute measurably to the potencies of ACTs.

Moreover, classical tyrosine kinase was not identified in the *Plasmodium falciparum* genome [74]. The use of a drug targeting human Syk, an enzyme expressed in erythrocytes, displays the potential advantage of preventing the selection of mutant resistant parasites.

## Figures and Tables

**Table 1:** Combination index value (CI) 24 and 48 h after treatment.

Syk Inhibitors (nM)	Dihydroartemisinin		Artesunate		Artemether	
	24 h	48 h	24 h	48 h	24 h	48 h
**P505-15 ^1^ (IC_50_ = 1 nM)**						
500	0.54 ± 0.03	0.75 ± 0.06	0.97 ± 0.03	0.55 ± 0.07	**0.87 ± 0.03**	0.83 ± 0.01
250	0.51 ± 0.01	0.71 ± 0.03	0.76 ± 0.08	0.51 ± 0.01	0.89 ± 0.01	0.65 ± 0.03
100	**0.42 ± 0.04**	0.55 ± 0.04	0.63 ± 0.03	0.61 ± 0.03	0.95 ± 0.01	0.63 ± 0.04
50	0.43 ± 0.05	**0.49 ± 0.01**	**0.58 ± 0.07**	**0.49 ± 0.05**	0.98 ± 0.04	**0.58 ± 0.01**
**Entospletinib ^1^ (IC_50_ = 7.7 nM)**						
500	0.52 ± 0.01	0.85 ± 0.06	0.87 ± 0.03	0.74 ± 0.04	1.01 ± 0.03	0.83 ± 0.04
250	**0.51 ± 0.07**	0.84 ± 0.07	0.73 ± 0.02	0.62 ± 0.01	**0.95 ± 0.05**	0.68 ± 0.04
100	0.77 ± 0.04	0.78 ± 0.03	0.66 ± 0.01	0.60 ± 0.02	0.98 ± 0.03	**0.63 ± 0.03**
50	0.82 ± 0.03	**0.69 ± 0.05**	**0.62 ± 0.02**	**0.55 ± 0.02**	1.04 ± 0.07	0.72 ± 0.04
**R406 ^1^ (IC_50_ = 41 nM)**						
500	**0.42 ± 0.04**	0.95 ± 0.06	0.82 ± 0.04	0.75 ± 0.02	0.85 ± 0.03	0.93 ± 0.04
250	0.46 ± 0.02	0.81 ± 0.03	**0.63 ± 0.01**	0.72 ± 0.01	0.82 ± 0.01	0.88 ± 0.04
100	0.50 ± 0.03	0.60 ± 0.04	0.76 ± 0.03	0.50 ± 0.02	**0.80 ± 0.04**	0.73 ± 0.03
50	0.60 ± 0.05	**0.58 ± 0.01**	0.92 ± 0.02	**0.44 ± 0.02**	0.82 ± 0.04	**0.72 ± 0.04**
**SYK II ^1^ (IC_50_ = 41 nM)**						
500	**0.51 ± 0.01**	0.92 ± 0.02	0.79 ± 0.04	0.89 ± 0.04	**0.97 ± 0.01**	0.73 ± 0.02
250	0.66 ± 0.01	0.93 ± 0.02	**0.55 ± 0.01**	0.99 ± 0.03	0.99 ± 0.03	0.58 ± 0.04
100	0.77 ± 0.06	0.52 ± 0.03	0.67 ± 0.04	0.96 ± 0.01	1.06 ± 0.05	**0.53 ± 0.05**
50	0.99 ± 0.04	**0.46 ± 0.05**	0.89 ± 0.02	**0.90 ± 0.04**	1.12 ± 0.01	0.62 ± 0.07
**Imatinib ^1^ (IC_50_ = 5 µM)**						
500	0.79 ± 0.02	0.78 ± 0.01	0.67 ± 0.03	0.75 ± 0.02	1.01 ± 0.02	0.83 ± 0.01
250	**0.73 ± 0.06**	0.57 ± 0.07	**0.61 ± 0.04**	0.96 ± 0.04	0.89 ± 0.03	0.78 ± 0.06
100	0.83 ± 0.01	0.58 ± 0.02	0.70 ± 0.01	0.64 ± 0.01	0.91 ± 0.01	**0.73 ± 0.04**
50	0.92 ± 0.05	**0.55 ± 0.05**	0.90 ± 0.02	**0.60 ± 0.02**	**0.85 ± 0.02**	0.82 ± 0.04
**Piceatannol ^1^ (IC_50_ = 10 µM)**						
500	0.57 ± 0.04	0.67 ± 0.02	0.77 ± 0.01	**0.65 ± 0.03**	0.95 ± 0.01	**0.73 ± 0.02**
250	**0.56 ± 0.01**	0.66 ± 0.04	**0.71 ± 0.03**	0.66 ± 0.07	0.78 ± 0.01	0.80 ± 0.01
100	0.68 ± 0.03	0.58 ± 0.05	0.80 ± 0.02	0.84 ± 0.02	0.85 ± 0.02	0.83 ± 0.04
50	0.79 ± 0.02	**0.49 ± 0.03**	085 ± 0.01	0.90 ± 0.04	**0.78 ± 0.03**	0.75 ± 0.01

Combination index (CI) (Chow–Talalay) of different Syk inhibitors (P505-15, R406, entospletinib, SYK II, piceatannol, and imatinib) at different concentrations (50–500 nM) in combination with different artemisinin derivatives (dihydroartemisinin, artesunate, and artemether) after 24 and 48 h of incubation: additive effect (C = 1), synergism (CI < 1), and antagonism (CI > 1). ^1^ IC_50_ on Syk catalytic subunit.

**Table 2:** Combination index value (CI) 24 h after treatment. Combination index (CI) (Chow–Talalay) of different SYK inhibitors (P505-15 and R406) at different concentrations (50–500 nM) in combination with different artemisinin derivatives (dihydroartemisinin and artesunate) using a fixed concentration of the iron chelator Deferasirox (DFX) after 24 and 48 h of incubation.

Syk Inhibitors (µM)	Deferasirox (50 µM)	
**P505-15**	**Dihydroartemisin**	**Artesunate**
500	1.08 ± 0.05	1.15 ± 0.07
250	1.05 ± 0.04	1.01 ± 0.09
100	1.08 ± 0.10	1.11 ± 0.03
50	1.29 ± 0.08	1.42 ± 0.05
**R406**		
500	1.03 ± 0.06	1.03 ± 0.02
250	0.96 ± 0.07	1.08 ± 0.04
100	1.17 ± 0.05	1.10 ± 0.06
50	1.29 ± 0.03	1.16 ± 0.08

## Supplementary Materials

The following are available online at https://www.mdpi.com/2076-3921/9/8/753/s1, Figure S1. Effect of RBC treatment with P505-15 (0.5 µM) and DHA (0.5 nM) on the accumulation of HMC after 12 h of incubation. Data are the average of five experiments ± SD; Figure S2 Effect of pRBC treatments with a representative Syk inhibitor (P505-15) (0.5 µM) and DHA (0.5 nM) on the accumulation of HMC after 3, 6, 12, and 24 h. Data are the average of five experiments ± SD. Significant differences to untreated pRBCs at * *p* < 0.05; ** *p* < 0.001; Figure S3. Isobolograms showing the interactions between Syk Inhibitors (P505-15 and imatinib) and dihydroartemisinin (DHA), after 24 h of incubation in the P. falciparum Palo Alto strain treated with G6PD-d red cells; Table S1. Combination index (CI) (Chow-Talalay) of different SYK inhibitors.
