# Supplementary material for: Syk Kinase Inhibitors Synergize with Artemisinins by Enhancing Oxidative Stress in Plasmodium falciparum-Parasitized Erythrocytes
Source: Antioxidants (Basel). 2020 Aug 14;9(8):753. doi: 10.3390/antiox9080753 (PMC7464437; doi:10.3390/antiox9080753)
Supplement: Supplementary file 1 [file antioxidants-09-00753-s001.pdf]

antioxidants

Supplementary materials

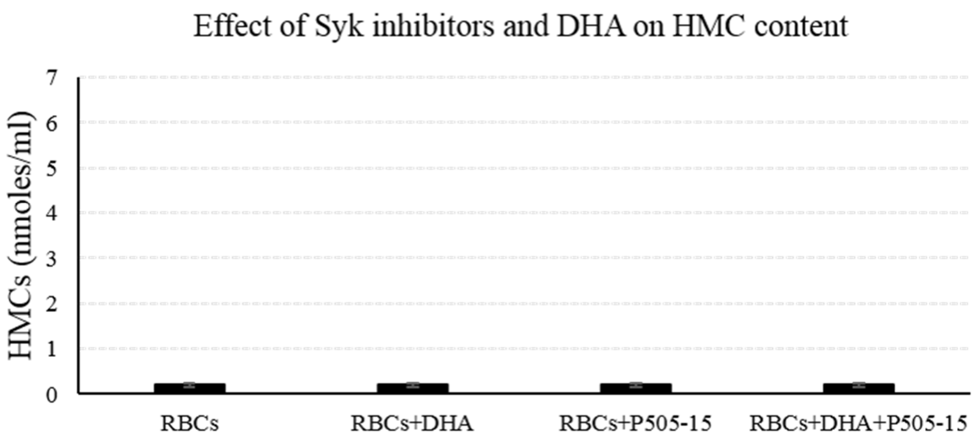

Figure S1. Effect of RBCs treatment with P505-15 (0.5 µM) and DHA (0.5 nM) on the accumulation of HMC after 12 h of incubation. Data are the average of 5 experiments ± S.D.

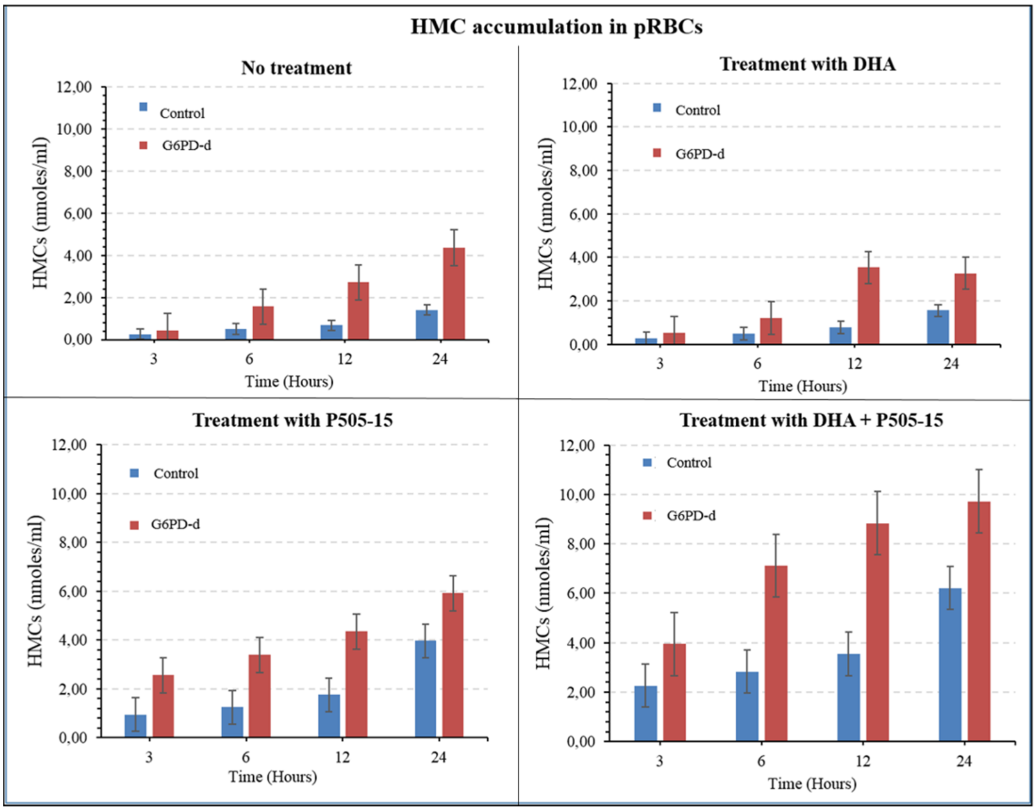

Figure S2.

Effect of pRBC treatments with a representative Syk inhibitor (P505-15) (0.5 µM) and DHA (0.5 nM) on the accumulation of HMC after 3,6,12 and 24 h in control and G6PD-d treated RBCs. Data are the average of 5 experiments ± S.D. Significant differences to untreated pRBCs at *$p < 0.05$; ** $p < 0.001$.

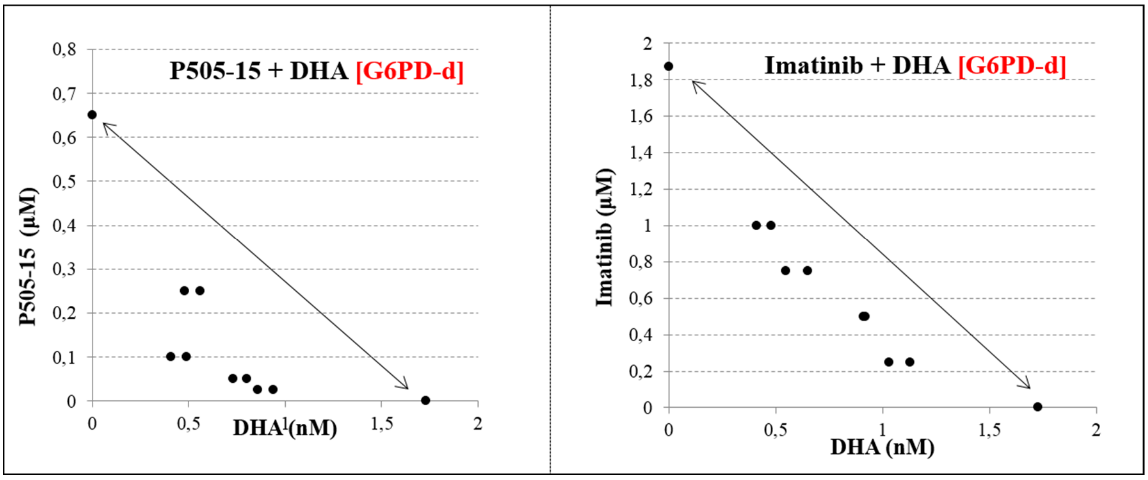

Figure S3. Isobolograms showing the interactions between Syk Inhibitors (P505-15 and Imatinib) with dihydroartemisinin (DHA), after 24 h of incubation in the *P. falciparum* Palo Alto strain treated with G6PD-d red cells.

Table 1. Combination index (CI) (Chow-Talalay) of different SYK inhibitors.

	Artesunate		Artemether	
	**24h**	**48h**	**24h**	**48h**
**P505-15 (IC_{50} = 1 nM)**	0.97±0.03	0.55±0.07	**0.87±0.03**	0.83±0.01
	0.76±0.08	0.51±0.01	0.89±0.01	0.65±0.03
	0.63±0.03	0.61±0.03	0.95±0.01	0.63±0.04
	**0.58±0.07**	**0.49±0.05**	0.98±0.04	**0.58±0.01**
**Entospletinib (IC_{50} = 1 nM)**	0.87±0.03	0.74±0.04	1.01±0.03	0.83±0.04
	0.73±0.02	0.62±0.01	**0.95±0.05**	0.68±0.04
	0.66±0.01	0.60±0.02	0.98±0.03	**0.63±0.03**
	**0.62±0.02**	**0.55±0.02**	1.04±0.07	0.72±0.04
**R406 (IC_{50} = 41 nM)**	0.82±0.04	0.75±0.02	0.85±0.03	0.93±0.04
	0.63±0.01	0.72±0.01	0.82±0.01	0.88±0.04
	**0.76±0.03**	0.50±0.02	**0.80±0.04**	0.73±0.03
	0.92±0.02	**0.44±0.02**	0.82±0.04	**0.72±0.04**
**SYK II (IC_{50} = 41 nM)**	0.79±0.04	**0.89±0.04**	**0.97±0.01**	0.73±0.02
	**0.55±0.01**	0.99±0.03	0.99±0.03	0.58±0.04
	0.67±0.04	0.96±0.01	1.06±0.05	**0.53±0.05**
	0.89±0.02	0.90±0.04	1.12±0.01	0.62±0.07
**Imatinib (IC_{50} = 5 µM)**	0.67±0.03	0.75±0.02	1.01±0.02	0.83±0.01
	**0.61±0.04**	0.96±0.04	0.89±0.03	0.78±0.06
	0.70±0.01	0.64±0.01	0.91±0.01	**0.73±0.04**
	0.90±0.02	**0.60±0.02**	**0.85±0.02**	0.82±0.04
**Piceatannol (IC_{50} = 10 µM)**	0.77±0.01	**0.65±0.03**	0.95±0.01	**0.73±0.02**
	**0.71±0.03**	0.66±0.07	**0.78±0.01**	0.80±0.01
	0.80±0.02	0.84±0.02	0.85±0.02	0.83±0.04
	085±0.01	0.90±0.04	0.78±0.03	0.75±0.01

SYK inhibitors (P505-15, R406, entospletinib, SYK II, piceatannol, and imatinib) at different concentrations (50–500 nM) in combination with different artemisinin derivatives (artesunate, and artemether) after 24 and 48 h of incubation. [additive effect (C = 1), synergism (CI < 1), and antagonism (CI > 1)].
